# Editorial: Update in trichology and hair transplantation

**DOI:** 10.3389/fmed.2026.1984488

**Published:** 2026-09-21

**Authors:** A. Gómez-Zubiaur, J. M. Ricart-Vayá, L. A. Sánchez-Dueñas, V. Velasco-Tamariz

**Affiliations:** 1 Trichology Unit, Ricart Medical Institute, Madrid, Spain; 2 Dermatology Department, Jalisco Dermatologic Institute “Dr. José Baba Rubio”, Zapopan, Mexico; 3 Department of Dermatology, Universitary Hospital 12 de Octubre and Morales-Raya Clinic, Madrid, Spain

**Keywords:** alopecia areata, androgenetic alopecia, artificial intelligence, diagnosis, frontal fibrosing alopecia, laser, senescent alopecia

## 1 Introduction

Hair is increasingly in the spotlight, as reflected by the economic growth of the global hair-care market. However, for dermatologists managing hair and scalp disorders, hair represents much more than a cosmetic issue. It performs relevant physiological functions, including thermoregulation and protection against solar ultraviolet radiation, while also playing a fundamental role in body image, personal identity, self-esteem, and psychological well-being.

Clinical practice clearly illustrates this importance. We can name some of our patients who suffer from alopecia areata totalis, severe androgenetic alopecia (AGA) or frontal fibrosing alopecia (FFA), or even squamous cell carcinoma developing on chronically sun-exposed areas of the scalp. This Research Topic is dedicated to these patients and to the many others attending trichology clinics. Its purpose is to improve the diagnosis and treatment of hair and scalp disorders through state-of-the-art assessment methods and therapeutic strategies supported by scientific evidence.

The expansion of the hair-care industry reflects the increasing relevance of hair health for companies, laboratories, and society. Nevertheless, physicians and researchers must place economic and commercial claims into perspective through scientific evidence ensuring that innovation translates into better patient care.

## 2 Overview of the research topic

This collection encompasses a broad spectrum of contemporary trichology, ranging from diagnostic methodology to medical and surgical treatments, with particular emphasis on disorders commonly encountered in clinical practice.

The diagnostic section explores state-of-the-art software incorporating artificial intelligence (AI), which is prompting reconsideration of historical alopecia classification systems (Sánchez-Dueñas et al.). Nevertheless, histopathological examination remains essential in selected cases to establish a definitive diagnosis (Gonzalez Cañete et al.).

Different laser technologies are also evaluated, both for telogen effluvium (TE), a frequent cause of hair shedding (Ruiz Dueñas et al.), and for reducing inflammation in FFA (Ferrer Guillén et al.). Another important focus is the impact of alopecia areata (AA) on quality of life and the instruments available to quantify the burden of this potentially stigmatizing disease (Muñoz-Barba et al.).

The collection additionally examines the evolving therapeutic landscape, including drugs undergoing clinical trials (Parra-Guerra et al.; Lv et al.) and emerging evidence concerning established treatments. These include minoxidil and antiandrogen therapies for AGA (Xia et al.; Larios-Cárdenas et al.) and contact immunotherapy for AA (Herz-Ruelas et al.).

A comprehensive approach to hair health should also integrate pharmacological treatment with appropriate dietary and lifestyle measures, while nutraceuticals may have a complementary role (Zhou et al.). Established adjuvant therapies such as platelet-rich plasma (PRP) continue to evolve as accumulating evidence helps optimize treatment protocols and potentially improve efficacy (Ghanem et al.).

Hair aging is also addressed, particularly progressive hair graying. Within the increasingly recognized framework of senescent alopecia (SA), certain active compounds may potentially modify aspects of the natural course of hair aging (Vega Diez et al.).

Hair transplantation represents another major pillar. Attention extends beyond surgical technique to the recognition and management of complications and unsatisfactory outcomes (Romera de Blas et al.), including correction of an inappropriate hairline (Gómez-Zubiaur et al.) and adverse effects associated with synthetic hair fibers (Scarano et al.). Careful postoperative follow-up is fundamental, including appropriate products before and after surgery. Scalp-cleansing shampoos may contribute to skin recovery and removal of postoperative scaling and crusting (Meyer et al.).

Together, these topics illustrate the comprehensive evidence-based approach required in modern trichology, integrating accurate diagnosis, innovative technologies, medical and surgical therapies, and holistic patient care.

## 3 Discussion of the contributing articles

AGA is a chronic condition affecting many women and even more men, explaining its substantial representation in this collection. Regarding diagnosis and staging, Sánchez-Dueñas et al. propose incorporating AI as a third pillar alongside trichoscopy and clinical assessment. AI-assisted follicular counting and image-analysis software could establish more precise criteria for disease onset and transitions between stages, particularly relevant for clinical trials. Standardization of metrics, images, and datasets will be essential to achieve this objective. The main treatments are minoxidil and 5-alpha-reductase inhibitors. Larios-Cárdenas et al. studied 67 men with male AGA divided into four treatment groups receiving minoxidil 1 mg, minoxidil 2.5 mg, minoxidil 5 mg, or dutasteride 0.5 mg for 24 weeks**.** No significant differences were identified in overall trichoscopic hair density, although individual parameters improved in different scalp regions and treatment groups. Clinical improvement occurred in all minoxidil groups and was greater with 2.5 and 5 mg, but was not observed in the dutasteride group. Treatment duration appears crucial when evaluating trichoscopic changes and determining the efficacy of 5-alpha-reductase inhibitors. In the Bayesian meta-analysis by de Xia et al. evaluating minoxidil as monotherapy or combined treatment, the highest-ranked combination was PRP + fibroblast growth factor + minoxidil, with a SUCRA of 93.06%**.** Among men, minoxidil + finasteride represented the best-ranked option (SUCRA 80.21%), whereas among women minoxidil + microneedling ranked highest (SUCRA 87.20%). PRP therefore remains prominent in AGA. According to the systematic review of three clinical trials by Ghanem et al., single-spin centrifugation produced better results, although statistical significance was not reached. Nutricosmetics continue to demonstrate moderate efficacy, according to Zhou et al., but available studies show substantial heterogeneity in design and supplement composition, particularly when comparing multi-ingredient formulations with isolated active ingredients.

According to Parra-Guerra et al., AA ranks second in registered clinical trials in recent years and has experienced the greatest growth. Its impact on quality of life is a major consideration. Muñoz-Barba et al. present the Spanish Alopecia Areata–Life Impact Questionnaire (SAALIQ). It comprises 10 questions covering emotional, functional, and social domains and provides a total score ranging from 10 to 40, with higher scores indicating poorer quality of life. Evaluation in 85 adults demonstrated good internal consistency and convergent validity. In children and adolescents, the psychological burden of AA may be even greater, reinforcing the need for effective treatments. JAK inhibitors have opened a new therapeutic horizon (Lv et al.), although some patients continue to respond effectively to combinations of traditional therapies, including contact immunotherapy, oral and intralesional corticosteroids, and minoxidil (Herz-Ruelas et al.).

Laser devices are increasingly relevant in trichology, although further evidence is required. Ruiz Dueñas et al. studied 10 women and 3 men with TE, of whom 69% had chronic and 31% acute disease. Patients received treatment with a 675-nm diode laser, using 0.5 W, 25-ms pulses and 20 min per session**,** for three sessions separated by four weeks**.** No adverse events occurred, while pruritus and trichodynia improved. Among the 11 patients with an initially positive hair-pull test, 8 (72%) became negative during follow-up, and none initially negative subsequently became positive. Nevertheless, the small sample and concomitant treatments require cautious interpretation.

The 308-nm excimer laser has also been successfully used for FFA. Ferrer Guillén et al. included 30 patients**,** 90% women, with a mean age of 65.2 years, treated for six months as adjunctive therapy without modifying previous treatments. These included corticosteroid mesotherapy in 90%**,** oral dutasteride in 86.7%**,** and topical corticosteroids in 83.3%**.** After a mean of 12 sessions and a cumulative dose of 5,254 mJ/cm^2^, frontotemporal recession/scarring stabilized in 53.3%**,** improved in 20%**,** and progressed in 26.7%**.** Perifollicular erythema improved by one grade in 83.3% of patients and perifollicular hyperkeratosis improved in 70%**.** Tolerability was high, with only four patients experiencing transient local erythema. Although treatment may focus on the frontal hairline, the remainder of the scalp should also be examined because FFA can involve occipital and retroauricular regions. González Cañete et al. additionally highlight the interparietal region. Histopathological lichenoid inflammatory changes were identified in all 12 patients studied**:** 6/12 were classified as FFA with interparietal involvement, 4/12 as fibrosing alopecia in pattern distribution (FAPD), and 2/12 as indeterminate cicatricial alopecia according to severity of the histological findings.

FFA predominantly affects peri. or postmenopausal women, and its diagnosis may overlap with physiological hair aging. Besides progressive graying, senescent alopecia includes generalized hair thinning, increased shedding, and marked xerosis of the scalp and hair shaft. Minoxidil has been proposed to counteract this involutional alopecia, together with newer molecules such as the proteoglycans decorin and versican, which have demonstrated potent antioxidant, anti-inflammatory, and pro-melanogenic activity *in vitro* (Vega Diez et al.).

Despite increasingly comprehensive pharmacological therapy, hair transplantation continues to occupy an important position in hair restoration, particularly for AGA. Growing demand has led to proliferation of centers worldwide, not all of which guarantee appropriate quality standards. Although transplantation is minimally invasive, it is methodologically demanding because meticulous execution is required at every stage. The final outcome depends on rigorous protocols encompassing appropriate design and realistic planning, extraction without transection or overharvesting, meticulous classification according to the number of hair shafts, placement without tissue or follicular-unit damage, and close postoperative follow-up. Even when procedures are performed correctly, complications can occur and must be appropriately recognized and managed, as summarized by Romera de Blas et al. Appropriate treatment of recipient skin and timely removal of postoperative crusts are essential for recovery. Certain active ingredients in shampoos may be beneficial, including clobetasol for its anti-inflammatory action and salicylic acid for its keratolytic effect (Meyer et al.). When transplantation produces an unsatisfactory result, corrective procedures should be considered. If the frontal hairline is excessively low, straight, or rounded, Gómez-Zubiaur et al. identify FUE extraction of unwanted follicular units as the corrective treatment of choice. Their multicenter study included 20 patients. The technique is complex and has higher-than-usual partial and total transection rates, so patients should be informed that a second procedure may be necessary to complete extraction. Nevertheless, satisfaction was high, with no visible scars in the frontal region six months after treatment**.** Finally, for patients without an adequate donor area or those unwilling to reduce its density, Scarano et al. propose synthetic hair fibers composed of polyamide, 0.08 mm in diameter and 160–460 mm in length, implanted at the level of the galea aponeurotica. Among 15 treated patients, only three developed mild inflammation and no foreign-body reactions occurred. However, total follow-up was only 12 months. Despite improvements in fiber composition, granulomatous reactions remain a relevant potential risk, and annual fiber loss ranges from 10% to 15%**.**

## 4 Conclusion

Appropriate support for patients affected by different forms of alopecia requires offering the best therapeutic options—not those associated with the highest cost, but those supported by solid and rigorous scientific evidence. Although the hair-care industry continues to expand, dermatologists specializing in trichology must remain guided by the principle of beneficence. This collection contributes in this direction and its central objective is to ensure that advances and innovation in trichology ultimately translate into meaningful improvements in patient care.

